# Preoperative Predictors of Ambulation Ability at Different Time Points after Total Hip Arthroplasty in Patients with Osteoarthritis

**DOI:** 10.1155/2014/861268

**Published:** 2014-08-10

**Authors:** Akiko Kamimura, Harutoshi Sakakima, Fumio Tsutsumi, Nobuhiko Sunahara

**Affiliations:** ^1^Course of Physical Therapy, School of Health Sciences, Faculty of Medicine, Kagoshima University, 8-35-1 Sakuragaoka, Kagoshima 890-8544, Japan; ^2^Kagoshima Physical Therapy Association, Kagoshima 897-0132, Japan; ^3^Red Cross Kagoshima Hospital, Kagoshima 891-0133, Japan; ^4^Department of Physical Therapy, Kyushu Nutrition Welfare University, Fukuoka 800-0298, Japan

## Abstract

The aims of this study were to identify the preoperative factors influencing ambulation ability at different postoperative time points after total hip arthroplasty (THA) and to examine the cutoff values of predictive preoperative factors by receiver operating characteristic (ROC) curves. Forty-eight women with unilateral THA were measured for hip extensor, hip abductor, and knee extensor muscle strength in both legs; hip pain (visual analog scale, VAS); and the Timed Up and Go (TUG) test pre- and postoperatively. Multiple regression analysis indicated that preoperative knee extensor strength (*β* = −0.379, *R* ^2^ = 0.409) at 3 weeks, hip abductor strength (*β* = −0.572, *R* ^2^ = 0.570) at 4 months, and age (*β* = 0.758, *R* ^2^ = 0.561) at 7 months were strongly associated with postoperative ambulation, measured using the TUG test. Optimal preoperative cutoff values for ambulation ability were 0.56 Nm/kg for knee extensor strength, 0.24 Nm/kg for hip abductor strength, and 73 years of age. Our results suggest that preoperative factors predicting ambulation ability vary by postoperative time point. Preoperative knee extensor strength, hip abductor strength, and age were useful predictors of ambulation ability at the early, middle, and late time points, respectively, after THA.

## 1. Introduction

Total hip arthroplasty (THA) is commonly performed in patients with hip osteoarthritis (OA). THA is effective in decreasing pain, increasing range of motion of the hip joint, increasing muscle strength and stability, and enabling patients with hip OA to return to their normal daily activities. The greatest amount of functional improvement is observed within 6 months postoperatively, with further improvements occurring for up to 2 years [1, 2]. The number of patients undergoing THA has increased due to rapid advancements in surgical techniques and other developments by healthcare professionals to achieve excellent outcomes, with early functional recovery and short hospital stays [3]. However, not all patients obtain the same amount of benefits from THA, and preoperative functional status appears to be an important predictor of the postoperative outcome [4–7].

As postoperative ambulation ability is an important factor for living an active life and independently performing daily activities, many patients wish to improve their postoperative ambulation ability. Several studies suggest that poor preoperative functional status is associated with poorer outcome after THA [4, 8]. Several parameters have been considered as possible preoperative predictors of ambulation outcome after THA in patients with OA. Preoperative factors associated with functional outcome include age, gender, physical function, level of pain, comorbid conditions, Medical Outcomes Study 36-Item Short Form Health Survey (SF-36) score, Western Ontario and McMaster Universities Osteoarthritis (WOMAC) score, and perception of self-efficacy [2, 4, 5, 9–13]. Based on these studies, we hypothesized that certain preoperative factors would influence changes in ambulation ability at different postoperative time points after THA.

Muscle atrophy occasionally occurs in patients with hip OA due to preoperative inactivity and may persist after THA; this muscle atrophy contributes to reduced ambulatory capacity and hip and knee muscle strength deficits [14, 15]. Hip abductor weakness on the operated side is reportedly a major risk factor for complications of THA surgery, such as joint instability or loosening [1, 16]. Greater preoperative knee extensor strength on the operated side is associated with better physical function at 12 weeks after THA [6]. However, the relationship between preoperative lower extremity muscle strength and postoperative ambulation ability at different postoperative time points after surgery is unclear.

In the present study, we investigated the recovery of lower extremity muscle strength, pain, and the Timed Up and Go (TUG) test results as indicators of ambulation ability at 3 weeks, 4 months, and 7 months after THA. Furthermore, we identified which preoperative factors were most likely to predict improvement in ambulation ability at each postoperative time point and examined the cutoff values for these factors by receiver operating characteristic (ROC) curves.

## 2. Materials and Methods

### 2.1. Participants

We performed a prospective observational study involving a preoperative inception cohort. A total of 74 patients underwent THA at Kagoshima Red Cross Hospital between August 2010 and November 2012. Our inclusion criteria were primary THA for unilateral hip OA, no symptoms in the contralateral hip joint, and informed consent to participate in this study. Patients undergoing arthroplasty for neoplastic disease or articular rheumatism were excluded. Forty-eight female patients who consented to participation were included in this study. The patient characteristics were mean age, 67.6 years (standard deviation [SD]: 10.2, range: 43–85); mean height, 150.4 cm (SD: 7.9, range: 134–170); mean body weight, 57.0 kg (SD: 9.6, range: 39–86); and mean body mass index, 25.2 kg/m^2^ (SD: 3.8, range: 18.6–37.5). All patients had undergone primary THA using a posterolateral approach with noncemented prostheses. For walking ability, all patients had a limp before surgery, including 5 with a mild limp, 21 with a severe limp, 20 with walking difficulty, and 2 who could not walk, indicating that the patients had severe hip OA. Most patients were only able to maintain an indoor walking speed and, if able to walk, required assistance device support.

All patients participated in a prescribed 3-week conventional rehabilitation program during hospitalization, according to the protocol of the Kagoshima Red Cross Hospital. This program consisted of joint range of motion, muscle strength, and functional exercises. Range of motion exercise of the knee or hip joint consisted of passive flexion, extension, abduction, and external rotation performed manually by a physical therapist. Muscle strength exercises were single-joint exercises for hip abduction/adduction, hip flexion/extension, and knee flexion/extension. Initially, active assisted and then active exercises without resistance were used. Later, exercises against progressive resistance were introduced, which was applied manually by a physical therapist. For hip abduction, resistance was applied by a Thera-Band fixed to the ankle with the patients in supine position. Partial weight bearing was initiated from the third postoperative day. Full weight bearing was initiated from postoperative day 14. Gait training started from the third postoperative day using a parallel bar, dependent on muscle pain. The walking distance on a level surface was increased gradually. All patients could walk independently with or without any device support at 3 weeks after surgery. Calf raise exercises, squatting, and single-leg standing exercises were performed to increase muscle strength and improve balance capacity. Physical therapy sessions lasted 1 hour per day. Outcome measurements were assessed at 3 weeks (at discharge) and at 4 and 7 months postoperatively. After discharge, participants presented as outpatients, at which time the measurements were recorded. All measurements were recorded by the same physical therapist. Informed consent was obtained from all patients, and this study was approved by the Ethics Committee of Kagoshima Red Cross Hospital.

### 2.2. Clinical Parameters

Leg muscle strength on the operated and nonoperated sides was measured using a hand-held dynamometer (HHD, Anima Co., *μ*-TasF1, Japan) during isometric contraction for 3 s with manual resistance. The HHD is a widely used, reliable, and valid instrument for measuring isometric peak force in studies of elderly patients or THA [6, 16, 17]. Positions chosen for testing were based on the previous literature [18–20] and considered patient safety for adherence to postoperative precautions after THA. To measure hip abductor strength, the subject lay in the supine position, with the hip and knee in neutral flexion/extension and the hips in neutral abduction/adduction. A force sensor was placed 5 cm proximal to the lateral epicondyle of the femur, and a dynamometer stabilizing belt passing around a bar secured the contralateral leg. During measurement of hip extensor strength, the subject was laid in the supine position, with the hip in 30° flexion, the knee in 50° flexion with a triangle stand under the knee, and the hip in neutral abduction/adduction. The force sensor was placed 5 cm proximal to the lateral epicondyle of the femur. During the measurement of knee extensor strength, the subject sat on the bed with the hip and knee at angles of 90°, the force sensor was placed 5 cm above the lateral malleolus, and a dynamometer stabilizing belt passing around a bar was secured behind the back legs of the bed. Successive measurements were performed 3 times, and the average score was used for analysis. Torque was calculated by multiplying strength by the lever arm and was expressed as a percentage of body weight (Nm/kg).

We evaluated hip pain using the visual analogue scale (VAS). The VAS score reflects the subjective degree of pain. Patients place a mark on a 100 mm line—complete absence of pain is indicated by a mark placed at the left edge at 0 mm, while maximum pain is indicated by a mark placed at the right edge at 100 mm.

To assess ambulation ability, we used the TUG test, including balancing and walking. The TUG test consists of standing up from an armless chair (seat height: 45 cm), walking in a straight line for 3 m, returning to the chair, and sitting down. Patients performed the test while wearing their own shoes and at a self-selected speed. We measured the TUG test performance time in seconds.

### 2.3. Patient Classification

Patients with a TUG test score > 13.5 seconds were classified as fallers in community-dwelling older adults [19]. Mean patient age was relatively elderly. Therefore, based on TUG test times, the patients were divided into the good (less than 13.5 s) and nongood (over 13.5 s) ambulation groups at 3 weeks, 4 months, and 7 months after THA.

### 2.4. Statistical Analysis

One-way analysis of variance (ANOVA) was used to compare clinical measurements. If significance was achieved, the Bonferroni test was performed to determine where the significant differences occurred. Student's *t*-test was used to determine differences in lower extremity muscle strength between the operated and nonoperated sides. The levels of association between the TUG test score and clinical parameters were examined using Pearson's rank correlation coefficients. When significant correlation coefficients were observed, stepwise multiple regression analysis was used to investigate the effect of the physical variables (preoperative hip abductor and extensor strength, knee extensor strength, VAS, TUG, age, and BMI), while using the TUG test results at different postoperative time points as the dependent variable. Mann-Whitney's *U* test was used to determine differences in preoperative parameters between the good and nongood ambulation groups. Subsequently, the discriminative properties of preoperative knee extensor strength, hip abductor strength, and age for ambulation ability were investigated by applying a receiver operating characteristic (ROC) curve. For this purpose, the area under the curve (AUC), sensitivity, and specificity were calculated. Statistical analyses were performed using SPSS (Version 20.0, SPSS Inc., Chicago, IL). For all tests, a *P* value of < 0.05 was considered statistically significant. All data are shown as mean ± standard deviation.

## 3. Results

### 3.1. Recovery of Clinical Parameters

Descriptive statistics of the pre- and postoperative values for lower extremity muscle strength, VAS, and TUG test score are indicated in Table 1. Hip extensor and abductor strength on the operated side improved significantly at 3 weeks postoperatively compared to the preoperative value. However, knee extensor strength at 3 weeks postoperatively was not significantly improved compared to the preoperative value. Hip and knee muscle strength on the operated side was significantly lower compared to that on the nonoperated side, both pre- and postoperatively. The VAS score significantly decreased at 3 weeks postoperatively (approximately 84%) compared with the preoperative value, and none of the patients reported hip pain at 4 and 7 months. The TUG test score significantly improved at 4 and 7 months postoperatively, compared to the preoperative value.

### 3.2. Preoperative Factors as Predictors of Ambulatory Ability

The results of Pearson's rank correlation analysis to assess the effect of preoperative factors on the postoperative TUG test score are shown in Table 2. At 3 weeks postoperatively (i.e., at discharge), the TUG test score was significantly correlated with all examined preoperative clinical parameters, except for hip extensor strength and VAS score. At 4 months postoperatively, the TUG test score was significantly correlated with all examined preoperative clinical parameters, except for VAS score. At 7 months postoperatively, the TUG test score was significantly correlated with preoperative knee extensor strength, TUG test score, age, and BMI.

These significant variables were entered into a stepwise multiple regression model as dependent variables, using the TUG test score for each postoperative phase as the dependent variable (Table 3). At 3 weeks postoperatively (at discharge), knee extensor strength, age, and BMI were significantly associated with the TUG test score. At 4 months postoperatively, hip abductor strength and age were significantly associated with the TUG test score. At 7 months postoperatively, age and BMI were significantly associated with the TUG test score.

**Figure 1:**
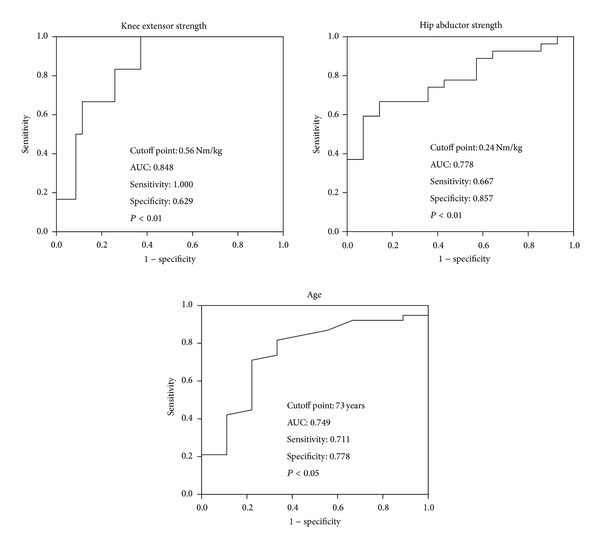
Receiver operating characteristic (ROC) curve of assessment of ambulation ability at 3 weeks, 4 months, and 7 months postoperatively, with preoperative knee extensor strength, hip abductor strength, and age, respectively.

Furthermore, the patients were divided into good and nongood ambulation groups to determine the cutoff values of these preoperative factors.  Table 4 shows age, BMI, and the clinical parameters of the 2 patient groups at different postoperative time points. Some preoperative clinical parameters were significantly decreased in the nongood ambulation group compared to the good ambulation group. ROC curves were then determined for the 3 associated preoperative factors for ambulation ability at 3 weeks and 4 and 7 months: preoperative knee extensor strength, hip abductor strength, and age, respectively. The ROC curve of each model was graphically displayed (Figure 1). Optimal preoperative cutoff values for ambulation ability of 0.56 Nm/kg knee extensor strength, 0.24 Nm/kg hip abductor strength, and 73 years of age were found, with area under the ROC curve (AUC) of 0.848 (95% confidence interval (CI): 0.715–0.981), 0.778 (95% CI: 0.637–0.919), and 0.749 (95% CI: 0.568–0.929), respectively. Sensitivity was 1.00 (95% CI: 1.000–1.000) for preoperative knee extensor strength, 0.667 (95% CI: 0.419–0.913) for preoperative hip abductor strength, and 0.711 (95% CI: 0.008–0.570) for preoperative age. Specificity was 0.69 (95% CI: 0.241–1.015) for preoperative knee extensor strength, 0.875 (95% CI: 0.725–0.989) for preoperative hip abductor strength, and 0.778 (95% CI: 0.072–0.327) for preoperative age.

## 4. Discussion

In this study, we identified that preoperative knee extensor strength, but not hip muscle strength, was strongly associated with ambulation ability, as measured by the TUG test, at 3 weeks postoperatively. Hip muscle strength is influenced by hip pain in patients with OA. Our results showed that hip muscle strength increased with decreasing hip pain but that knee extensor strength did not significantly increase at 3 weeks postoperatively. Knee extensor strength decreased in patients with hip OA and was associated with motor performance [21, 22]. Most patients with THA undergo a short rehabilitation program and are discharged immediately after regaining minimal ambulatory ability, such as walking with a cane. Therefore, preoperative knee extensor strength may be considered as a predictor of ambulation ability at an early phase after surgery. This study suggests that preoperative knee extensor strength may be useful as a predictor of ambulation ability at approximately the first month after THA.

Our analysis showed that preoperative hip abductor strength, but not knee and hip extensor strength, was strongly associated with ambulation ability at 4 months postoperatively. Our patients did not experience hip pain at this time and showed progressive improvement in clinical parameters from 3 weeks to 4 and 7 months postoperatively. The TUG test score is influenced by the degree of hip function, as well as pain, leg muscle strength, and range of motion. Loss of abduction power is a common problem for patients with OA and is a major risk factor for complications of THA surgery. Weaker hip muscles, especially hip abductor muscles, on the affected site in patients with THA may also contribute to poor trunk control while walking [16]. Hip abductor strength may have a long-term effect on gait function. Therefore, preoperative hip abductor muscle strength can be used as a predictor of ambulation ability at 4 months after THA, in conjunction with decreased pain and increased hip function. However, our analysis revealed that preoperative age, but not hip and knee muscle strength, was significantly associated with ambulation ability at 7 months postoperatively. Our analysis showed that age and BMI were associated with the TUG test score at all postoperative time points. It is possible to identify patients with THA who are at risk of an unsuccessful outcome by using variables such as BMI, age, and sex [13]. Our findings suggest that age may also be an important predictor of ambulation ability when muscle strength and leg function had almost improved after THA.

The TUG test is widely used to assess functional mobility in elderly people. Preoperative lower extremity muscle strength and the TUG test scores have been found to be sensitive to functional changes in patients following THA [6, 23]. Therefore, we used the TUG test to assess ambulation ability. Nankaku et al. [23] reported that patients with a preoperative TUG test score of less than 10 seconds are likely to walk without an assistive device at 6 months after THA. In this study, few patients had a preoperative TUG test score less than 10 seconds because of severe hip pain. Our patients with end-stage OA had a lower TUG test score than patients in other studies [19, 23]. Therefore, we used the TUG test score classification for elderly people.

Individuals with hip OA are less physically active than their healthy peers [19]. Lower level of physical activities in individuals with hip OA was evident in diminished functional ability and was a risk factor for future physical limitation [24, 25]. Because our patients had less physical activity and activity of daily life preoperatively, they may have had severe disuse atrophy of leg muscles as well as severe hip pain. Therefore, the values of preoperative hip and knee muscle strength were low compared with other studies on Japanese women undergoing THA. Our patients might have had not only severe hip pain, but also a sense of fear during the leg muscle strength test. Due to the hip pain, we did not give positive verbal encouragement during the muscle strength test. However, all patients had improved clinical parameters after surgery, including decreased hip pain. The improved clinical parameters may be reflected because of the low preoperative values in the patients with less physical activity and disuse leg muscle atrophy.

The present study further suggests that preoperative knee extensor strength, hip abductor strength, and age, with cutoff points of 0.56 Nm/kg, 0.24 Nm/kg, and 73 years, may be a good predictive factor for ambulation ability at 3 weeks and 4 and 7 months after THA. Most patients with THA are anxious about the postoperative recovery of their ability to walk before surgery. It is thus very helpful for them to be able to predict their ambulation ability after THA from preoperative lower extremity muscle strength even if this study included severe OA patients with less preoperative physical activity. The TUG test scores were not significantly correlated with the preoperative VAS scores in the present study. Therefore, other parameters may be needed to investigate ambulation ability, such as 6 min walking distance or biomechanical analysis, in patients with THA.

Preoperative exercise programs are feasible and effective in improving early recovery of physical function after THA [7]. Gait speed is affected by knee extension and hip abduction muscle strength [26]. In this study, preoperative knee extensor and hip abductor strength were correlated with ambulation ability at 3 weeks and 4 months postoperatively. Therefore, preoperative exercise programs should target not only hip abductor muscle strengthening, but also knee muscle strengthening to improve ambulation ability. Generally, knee extension and hip abduction muscle strength are very important for ambulation ability following THA. It is important for physical therapists to know that preoperative predictors for ambulation ability vary by postoperative time points. Intensive preoperative exercise programs and instruction of physical therapy after surgery may be necessary for the patients who are below cutoff values in the present study.

The present study has certain limitations. First, we used a small sample size. Second, we measured only 3 muscle groups: hip abductor and extensor muscles and knee extensor muscles. The hip extensor, flexor, and abductor muscle strength as well as the knee extensor muscle strength have been found to be sensitive to OA, rather than the hip adductor and knee flexor strength [27]. In addition, as our subjects were operated on using the posterolateral approach, this surgery may cause a greater decrease in hip extensor strength than hip flexor strength. Therefore, we selected the hip abductor and extensor and knee extensor muscles to assess patients who underwent THA in the present study. Third, no long-term follow-up measurements were recorded. Most of the patients with THA are discharged within 3-4 weeks postoperatively, and the greatest amount of functional improvement after THA is observed within 6 months postoperatively [16, 17, 27]. Therefore, the final measurement in our study was performed at 7 months postoperatively. Despite these limitations, this study yields valuable findings that will help predict ambulation ability after THA in patients with hip OA.

## 5. Conclusion

This study suggests that preoperative factors predicting ambulation ability vary from early to late postoperative time points, and preoperative cutoff values of 0.56 Nm/kg for knee extensor strength, 0.24 Nm/kg for hip abductor strength, and 73 years of age were reliable predictors of ambulation ability at 3 weeks, 4 months, and 7 months, respectively, after THA in patients with OA.

## Figures and Tables

**Table 1:** Descriptive statistics for clinical measurements obtained pre- and postoperatively.

	Preoperative	3 weeks	4 months	7 months
Hip extensors (Nm/kg)				
Operated side	0.30 ± 0.12^†^	0.38 ± 0.13^∗†^	0.59 ± 0.13^∗†^	0.59 ± 0.30^∗†^
Nonoperated side	0.38 ± 0.13	0.46 ± 0.15	0.62 ± 0.12*	0.63 ± 0.04*
Hip abductors (Nm/kg)				
Operated side	0.24 ± 0.11^†^	0.32 ± 0.1^∗†^	0.50 ± 0.10^∗†^	0.51 ± 0.12^∗†^
Nonoperated side	0.34 ± 0.12	0.39 ± 0.10	0.54 ± 0.09*	0.57 ± 0.15*
Knee extensors (Nm/kg)				
Operated side	0.54 ± 0.28^†^	0.51 ± 0.22^†^	0.76 ± 0.17^∗†^	0.80 ± 0.08^∗†^
Nonoperated side	0.64 ± 0.24	0.64 ± 0.23	0.85 ± 0.21*	0.93 ± 0.13*
VAS score (mm)	48.1 ± 24.4	7.5 ± 10.6*	0*	0*
TUG test score (s)	23.2 ± 15.4	19.5 ± 7.8	11.9 ± 3.4*	10.7 ± 2.3*

Values are presented as mean ± SD. VAS: visual analog scale. TUG test: Timed Up and Go test. *P < 0.05, for comparisons with preoperative values. ^†^ *P* < 0.05 for comparisons with the nonoperated side.

**Table 2:** Pearson's rank correlation coefficients between clinical parameters and ambulation ability (measured using the TUG test) after THA.

	3 weeks postoperatively	4 months postoperatively	7 months postoperatively
Hip			
Extensors	−0.292	−0.476∗∗	−0.437
Abductors	−0.382∗	−0.612∗∗	−0.131
Knee extensors	−0.483∗∗	−0.579∗∗	−0.598∗∗
VAS score	−0.002	0.006	0.281
TUG test score	0.480∗∗	0.492∗∗	0.668∗∗
Age	0.436∗∗	0.638∗∗	0.612∗∗
BMI	0.393∗∗	0.404∗	0.390∗

Values are correlation coefficients. VAS: visual analog scale. TUG: Timed Up and Go test. BMI: body mass index. *P < 0.05, **P < 0.01.

**Table 3:** Multiple regression analysis using the TUG test score for ambulation ability at 3 weeks, 4 months, and 7 months after THA.

	3 weeks postoperatively	4 months postoperatively	7 months postoperatively
Hip			
Extensors		ns	
Abductors	ns	*β* = −0.572**	
Knee extensors	*β* = −0.379**	ns	ns
TUG test score	ns	ns	ns
Age	*β* = 0.334**	*β* = 0.444**	*β* = 0.758**
BMI	*β* = 0.314**	ns	*β* = 0.363*
Model fit	*R* ^2^ = 0.409	*R* ^2^ = 0.570	*R* ^2^ = 0.561
	*F* = 9.31	*F* = 23.7	*F* = 11.9
	*P* < 0.001	*P* < 0.001	*P* < 0.001

*β* is the standardization coefficient. *R* ^2^ is the coefficient of determination. VAS: visual analog scale. TUG: Timed Up and Go test. BMI: body mass index. *P < 0.05, **P < 0.01.

**Table 4:** Preoperative clinical parameters of patients with good and nongood ambulation ability at 3 weeks, 4 months, and 7 months after THA.

	3 weeks postoperatively		4 months postoperatively		7 months postoperatively	
	Good (*n* = 7)	Nongood (*n* = 41)	Good (*n* = 31)	Nongood (*n* = 17)	Good (*n* = 39)	Nongood (*n* = 9)
Hip						
Extensors (Nm/kg)	0.41 ± 0.12*	0.28 ± 0.11	0.33 ± 0.12*	0.24 ± 0.11	0.30 ± 0.11	0.28 ± 0.16
Abductors (Nm/kg)	0.34 ± 0.09	0.22 ± 0.10	0.28 ± 0.10*	0.17 ± 0.08	0.25 ± 0.10	0.18 ± 0.13
Knee extensors (Nm/kg)	0.84 ± 0.24*	0.49 ± 0.25	0.62 ± 0.27*	0.40 ± 0.23	0.55 ± 0.25	0.53 ± 0.42
VAS score (mm)	34.0 ± 29.4	50.5 ± 23.6	48.0 ± 24.5	48.3 ± 26.4	46.6 ± 24.4	62.1 ± 17.3
TUG test score (s)	13.1 ± 2.6*	25.2 ± 16.1	19.6 ± 12.1*	30.7 ± 19.0	21.8 ± 12.2	31.0 ± 27.1
Age (years)	59.6 ± 6.4*	69.0 ± 10.1	64.5 ± 10.3*	73.3 ± 7.2	65.9 ± 10.0*	73.8 ± 8.7
BMI (kg/m^2^)	24.1 ± 1.3	25.4 ± 4.1	25.0 ± 3.9	25.4 ± 3.8	25.0 ± 4.0	25.9 ± 3.5

Values are presented as mean ± SD. VAS: visual analog scale. TUG test: Timed Up and Go test. *P < 0.05 for comparisons with nongood values.

## References

[1] Long WT, Dorr LD, Healy B, Perry J (1993). Functional recovery of noncemented total hip arthroplasty. *Clinical Orthopaedics and Related Research*.

[2] Kennedy DM, Hanna SE, Stratford PW, Wessel J, Gollish JD (2006). Preoperative function and gender predict pattern of functional recovery after hip and knee arthroplasty. *The Journal of Arthroplasty*.

[3] Ibrahim MS, Khan MA, Nizam I, Haddad FS (2013). Peri-operative interventions producing better functional outcomes and enhanced recovery following total hip and knee arthroplasty: an evidence-based review. *BMC Medicine*.

[4] Fortin PR, Clarke AE, Joseph L (1999). Outcomes of total hip and knee replacement: preoperative functional status predicts outcomes at six months after surgery. *Arthritis and Rheumatism*.

[5] Nilsdotter A-K, Petersson IF, Roos EM, Lohmander LS (2003). Predictors of patient relevant outcome after total hip replacement for osteoarthritis: a prospective study. *Annals of the Rheumatic Diseases*.

[6] Holstege MS, Lindeboom R, Lucas C (2011). Preoperative quadriceps strength as a predictor for short-term functional outcome after total hip replacement. *Archives of Physical Medicine and Rehabilitation*.

[7] Oosting E, Jans MP, Dronkers JJ (2012). Preoperative home-based physical therapy versus usual care to improve functional health of frail older adults scheduled for elective total hip arthroplasty: a pilot randomized controlled trial. *Archives of Physical Medicine and Rehabilitation*.

[8] Holtzman J, Saleh K, Kane R (2002). Effect of baseline functional status and pain on outcomes of total hip arthroplasty. *Journal of Bone and Joint Surgery A*.

[9] Dohnke B, Knäuper B, Müller-Fahrnow W (2005). Perceived self-efficacy gained from, and health effects of, a rehabilitation program after hip joint replacement. *Arthritis and Rheumatism*.

[10] Röder C, Staub LP, Eggli S, Dietrich D, Busato A, Müller U (2007). Influence of preoperative functional status on outcome after total hip arthroplasty. *The Journal of Bone and Joint Surgery A*.

[11] Quintana JM, Escobar A, Aguirre U, Lafuente I, Arenaza JC (2009). Predictors of health-related quality-of-life change after total hip arthroplasty. *Clinical Orthopaedics and Related Research*.

[12] Clement ND, MacDonald D, Howie CR, Biant LC (2011). The outcome of primary total hip and knee arthroplasty in patients aged 80 years or more. *Journal of Bone and Joint Surgery B*.

[13] Slaven EJ (2012). Prediction of functional outcome at six months following total hip arthroplasty. *Physical Therapy*.

[14] Rasch A, Byström AH, Dalen N, Berg HE (2007). Reduced muscle radiological density, cross-sectional area, and strength of major hip and knee muscles in 22 patients with hip osteoarthritis. *Acta Orthopaedica*.

[15] Rasch A, Byström AH, Dalén N, Martinez-Carranza N, Berg HE (2009). Persisting muscle atrophy two years after replacement of the hip. *The Journal of Bone and Joint Surgery B*.

[16] Nankaku M, Tsuboyama T, Kakinoki R, Akiyama H, Nakamura T (2011). Prediction of ambulation ability following total hip arthroplasty. *Journal of Orthopaedic Science*.

[17] Kamimura A, Sakakima H, Miyazaki M (2013). Pelvic inclination angle and hip abductor muscle strength after total hip arthroplasty. *Journal of Physical Therapy Science*.

[18] Thorborg K, Bandholm T, Hölmich P (2013). Hip- and knee-strength assessments using a hand-held dynamometer with external belt-fixation are inter-tester reliable. *Knee Surgery, Sports Traumatology, Arthroscopy*.

[19] Judd DL, Thomas AC, Dayton MR, Stevens-Lapsley JE (2014). Strength and functional deficits in individuals with hip osteoarthritis compared to healthy, older adults. *Disability and Rehabilitation*.

[20] Bohannon RW, Bubela DJ, Wang YC, Magasi SR, Gershon RC (2011). Adequacy of belt-stabilized testing of knee extension strength. *Journal of Strength and Conditioning Research*.

[21] Lin Y-, Davey RC, Cochrane T (2001). Tests for physical function of the elderly with knee and hip osteoarthritis. *Scandinavian Journal of Medicine and Science in Sports*.

[22] Suetta C, Aagaard P, Magnusson SP (2007). Muscle size, neuromuscular activation, and rapid force characteristics in elderly men and women: effects of unilateral long-term disuse due to hip-osteoarthritis. *Journal of Applied Physiology*.

[23] Nankaku M, Tsuboyama T, Akiyama H (2013). Preoperative prediction of ambulatory status at 6 months after total hip arthroplasty. *Physical Therapy*.

[24] Veenhof C, Huisman PA, Barten JA, Takken T, Pisters MF (2012). Factors associated with physical activity in patients with osteoarthritis of the hip or knee: a systematic review. *Osteoarthritis and Cartilage*.

[25] Pisters MF, Veenhof C, van Dijk GM, Heymans MW, Twisk JWR, Dekker J (2012). The course of limitations in activities over 5 years in patients with knee and hip osteoarthritis with moderate functional limitations: Risk factors for future functional decline. *Osteoarthritis and Cartilage*.

[26] Bohannon RW (1997). Comfortable and maximum walking speed of adults aged 20-79 years: reference values and determinants. *Age and Ageing*.

[27] Rasch A, Dalén N, Berg HE (2010). Muscle strength, gait, and balance in 20 patients with hip osteoarthritis followed for 2 years after THA. *Acta Orthopaedica*.

